# Selection of possible signature peptides for the detection of bovine lactoferrin in infant formulas by LC-MS/MS

**DOI:** 10.1371/journal.pone.0184152

**Published:** 2017-09-19

**Authors:** Mingmei Yuan, Cong Feng, Shouyun Wang, Weiwei Zhang, Mo Chen, Hong Jiang, Xuesong Feng

**Affiliations:** 1 Department of Pharmaceutical Analysis, School of pharmacy, China Medical University, Shenyang, PR China; 2 Department of Health Laboratory Technology, School of Public Health, China Medical University, Shenyang, PR China; Gaziosmanpasa Universitesi, TURKEY

## Abstract

An LC-MS/MS assay based on a signature peptide was developed and fully validated for the quantitation of bovine lactoferrin in infant formulas. Three unreported signature peptides were derived and identified from the tryptic peptides of bovine lactoferrin. The peptide ETTVFENLPEK was used for quantification based on assay performance. The blank matrix camel milk powder and bovine lactoferrin protein standards were mixed and spiked with stable isotope-labeled internal standard to establish a calibration curve. The established method was extensively validated by determining the linearity (R^2^ > 0.999), sensitivity (limit of quantitation, 0.16 mg/100 g), recovery (83.1–91.6%), precision (RSD < 5.4%) and repeatability (RSD < 7.7%). To validate the applicability of the method, four different brands of infant formulas in China were analysed. The acquired contents of bovine lactoferrin were 52.60–150.56 mg/100 g.

## Introduction

Lactoferrin is an 80 kDa iron-binding glycoprotein of the transferrin family that is widely distributed in colostrum and milk [1,2]. It has been reported that lactoferrin has various physiologic and nutritional functionalities. As part of the innate immune system, lactoferrin possesses a wide spectrum of important biological functions, including ion metabolism, antimicrobial, antiviral, antioxidation, anti-inflammatory, and anticancer functions, wound healing, and immune modulation [3–7]. A clinical study on bovine lactoferrin added to infant formula showed a reduction in upper respiratory disease in infants 6–12 months of age [8]. The lactoferrin content in milk varies between different mammalian species and, within a given species, between lactation periods [3]. Particularly high concentrations of lactoferrin are found in human breast milk [9], while the content of lactoferrin in cow's milk is relatively low. It is worth noting that there is a very high (77%) structural homology between bovine lactoferrin (extracted and purified from cow milk) and human lactoferrin (either extracted from colostrum or produced via recombinant engineering) [10]. Infant formula supplementation with bovine lactoferrin-rich whey protein concentrates will play a significant role in functional improvement, which makes the protein composition close to that in human milk. It has been granted the “generally recognized as safe” status by the Food and Drug Administration [11].

Currently, a variety of methods for measuring lactoferrin have been described, including immunochemical techniques, enzyme-linked immunosorbant assays (ELISA)[12], gel electrophoresis (GE) [13], high-performance liquid chromatography (HPLC) [14], capillary electrophoresis (CE) [15] and liquid chromatography coupled to tandem mass spectrometry (LC-MS/MS)[16]. Of these methods, LC-MS/MS is a new gradually developed technology in the field of protein quantification in complex biological matrices [17,18]. LC-MS/MS as a promising alternative provides wide dynamic range, excellent selectivity, good accuracy and precision compared with conventional protein quantification methods on the analysis of compounds in complex biological matrices. It has been used by other researchers to examine the milk proteome and has resulted in the identification of a number of proteins with surrogate peptides including α-lactalbumin, β-lactoglobulin, casein proteins (α-, β- and κ-casein) and lactoferrin [19–21].

In this paper, the study aimed at establishing and validating a reliable LC-MS/MS method for the quantification of bovine lactoferrin based on multiple reaction monitoring (MRM) mode in infant formulas. This paper describes details of screening process of signature peptides used for the qualitative and absolutely quantitative analysis of bovine lactoferrin. Moreover, the methodological evaluation was carried out and the blank matrix was screened to achieve more accurate quantification of bovine lactoferrin in infant formulas. Finally, the contents of bovine lactoferrin in various infant formulas in China were determined using the developed LC-MS/MS method.

## Materials and methods

### Chemicals

Ammonium bicarbonate (NH_4_HCO_3_), dithiotheritol (DTT), iodoacetamide (IAA), formic acid (FA) and acetonitrile (ACN) were obtained from Sigma-Aldrich (St. Louis, MO, USA). All the reagents used were analytical or HPLC grade. Lactoferrin isolated from bovine milk (purity ≥85%) were purchased from Sigma-Aldrich. Trypsin (sequencing grade modified, V511 20 μg lyophilized enzyme) was purchased from Promega (Madison, WI, USA). Camel milk powder used as the control matrix was obtained from Al Ain Dairy (UAE, Dubai). All the peptide standards were chemically synthesized by SynPeptide Co., Ltd. (Shanghai, China) with purity above 95%. The internal standard (IS) peptide was the synthetic peptide containing a stable isotopically labelled amino acid ([^13^C_6_, ^15^N_2_] lysine). The stock solutions of the synthetic peptides were prepared using 20% acetonitrile/water solution. Ultrapure water was prepared using a Milli-Q Gradient A-10 purification system (Millipore, Bedford, MA, USA) during all the experiments. All chemical agents were prepared with 100 mM NH_4_HCO_3_ without further purification. Infant formulas were obtained at local retailers.

### Preparation of calibration standards and Quality Control Samples (QCs)

Camel milk powder was used as the blank biological matrix without bovine lactoferrin throughout this investigation. It was dissolved with 50 mM NH_4_HCO_3_ and diluted to the final concentration of 10 mg mL^-1^. Calibration standards (1, 5, 10, 20, 50, and 100 nM) and QCs (3.5, 15, and 75 nM) were prepared from the stock solutions of bovine lactoferrin (1 mM) by serial dilutions with blank matrix solution. The lyophilized synthetic isotopically labelled peptide ETTVFENLPEK* containing [^13^C_6_, ^15^N_2_] lysine (heavy peptide, molecular weight of 657.8^2+^) was prepared at a concentration of 2 mM and stored at -80°C. The heavy peptide served as an internal standard to construct the calibration curve. All working standard solutions were prepared by diluting stock solutions with the blank matrix immediately before use.

### Preparation of samples

Prior to trypsin digestion, 0.1 g of infant formulas were dissolved with 10 ml deionized water. Aliquots of 200 μL sample solutions (standards, QCs, blanks, or study samples) were spiked with 20 μL of 1 μM SIL-IS ETTVFENLPEK* working solution, except for blank matrix samples to which 20 μL of deionized water was added. The mixed solution was further reduced with 100 μL of 100 mM DTT solution kept for 60 min at 60°C. Cysteine residues were alkylated by adding 200 μL of 100 mM IAA solution and the samples were incubated in the dark for 30 min at 30°C in a preheated thermomixer (1000 rpm). Subsequently, the mixture was digested by adding 200 μL of 1 mg mL^-1^ reconstituted trypsin solution prepared in 25 mM NH_4_HCO_3_ and incubated overnight at 37°C in a preheated thermomixer at 1000 rpm. The reaction was stopped by adding 200 μL of quenching solvent (10% formic acid/water solution, v/v). The final tryptic samples were centrifuged at 12000g for 10 min at 4°C. The supernatant was passed through a 0.22 μm nylon filter and transferred to a clean collection Eppendorf tube for LC-MS/MS analysis.

### Liquid chromatography

Peptide identification was done with a 1200 Series LC module (Agilent Technologies, Santa Clara, CA, USA) including a vacuum degasser, two quaternary pumps, a temperature-controlled Agilent 1290 Infinity autosampler kept at 10°C and a thermostated column compartment kept at 40°C. The LC system was controlled by the Agilent Mass Hunter Workstation Data Acquisition software for programming samples. The analytical column was an Agilent ZORBAX SB-C18 column (50 mm length, 2.1 mm internal diameter, 1.8 μm particle size). The separation was carried out by injecting 5 μL of samples under a binary elution gradient operated at a flow-rate of 0.4 mL min^-1^. The used mobile phase consisted of solvent A (0.1% formic acid in MilliQ water) and solvent B (0.1% formic acid in ACN). The total run time for each injection was 5 min using a gradient elution. The LC run started with 10% B for 0.2 min, followed by a gradient to 50% B in 2.8 min and another gradient to 95% B in 0.2 min. The column was washed using 95% B for 0.2 min and then returned to 10% B in 0.2 min, re-equilibrating for 1.1 min before the next injection.

### Mass spectrometry

The MS detection system consisted of an Agilent Technologies 6420 triple quadrupole with ESI in positive ionization mode for the search and identification of signature peptides from tryptic hydrolysates. The ionization source conditions were set as follows: capillary voltage, 4.0 kV; source temperature, 100°C; desolvation gas (N_2_), 350°C; and gas flow 10 L min^-1^. The nebulizer pressure was set at 50 PSI to ensure sufficient nebulization for the chromatographic conditions. Collision gas was highly pure nitrogen. Quantitation was performed under MRM mode. For each MRM transition, a dwell time of 200 ms was chosen, and the pause between mass ranges was 5 ms. The Mass Hunter Workstation Quantitative Analysis software was applied to calculate the integration peak area of the MRM transitions of each analyte. The online BLAST search in UniProt (www.uniprot.org) and NCBI (www.ncbi.nhn.nih.gov) were used for evaluating the specificity of the signature peptide selected for bovine lactoferrin. All LC-MS measurements were performed in triplicate.

### Assay validation and sample analysis

The established method was evaluated in accordance with international guideline[22] for bioanalytical method validation. In order to ensure the accuracy of method, we validated limit of quantification, linearity, specificity, repeatability, recovery and precision (intra- and inter-day). The calibration curve was prepared by spiking bovine lactoferrin standards into the blank matrices. Subsequently, the validated method was applied to measure concentrations of bovine lactoferrin in different infant formulas. Data were expressed as mg/100 g.

## Results and discussion

### Selecting an optimal signature peptide for bovine lactoferrin

**Fig 1:**
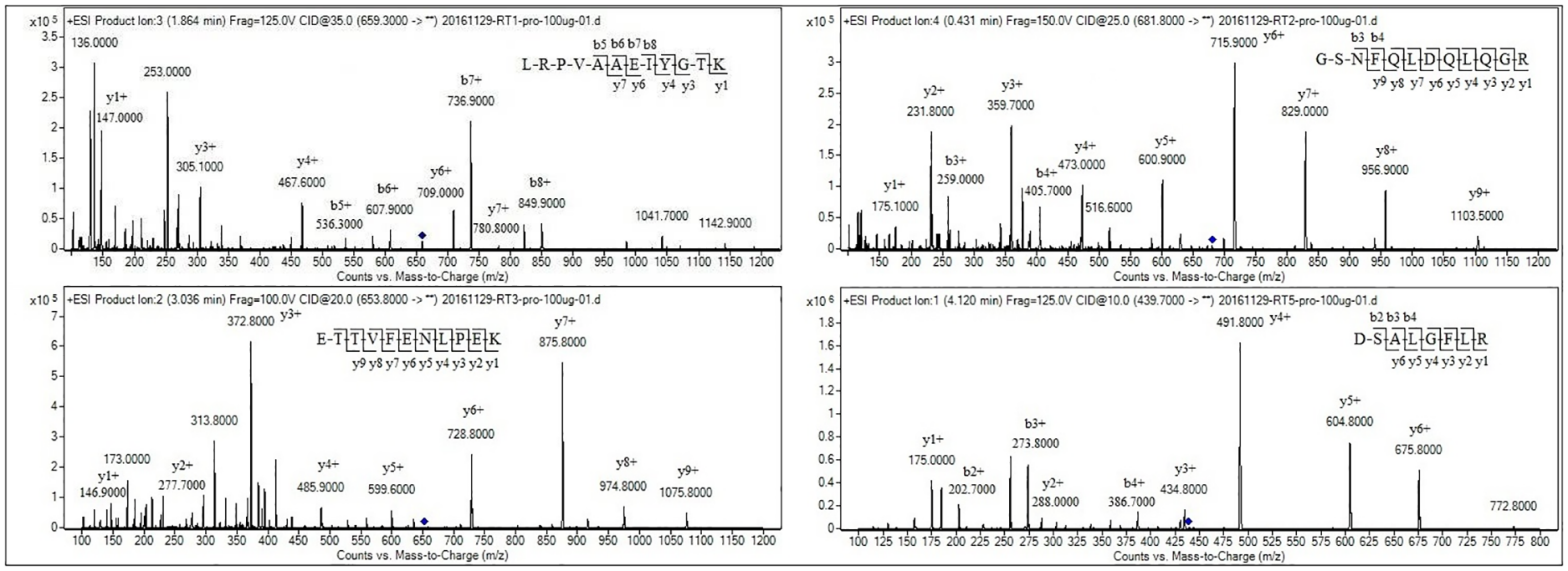
Product ion spectrums for the four peptide candidates with identified b and y ions.

One of the most critical steps for successfully establishing LC-MS/MS approaches for protein quantitation is selecting suitable signature peptides from the tryptic hydrolysates. The theoretical tryptic peptides of bovine lactoferrin (UniProt ID: P24627, S1 File) were obtained by computational prediction using Skyline software (Version 19). The endogenous tryptic peptides derived from digested bovine lactoferrin standard were separated by LC and confirmed by MS in MRM mode. Twenty peptides were verified after comparing in silico prediction peptides with actual tryptic peptides (S1 File). These peptides obtained in tryptic hydrolysates of bovine lactoferrin served as candidate signature peptides. Further screening was essential in accordance with principles of the selection procedure for signature peptides [23] for the sake of selecting the optimal signature peptide. Multiple signature peptides are preferred to optimize the various critical factors and to reduce the risk of falsely quantifying results. Eventually, four tryptic peptides reproducibly detected were selected from a list of moderate to high abundance peptides by systematically analysis of measured peptides. The four peptides with appropriate length between 8 and 15 amino acids selected in this procedure had good stability, specificity, chromatography and mass spectrometry behaviour. The double charged fragment ions of the four peptides were m/z 659.3[92–103], 681.8[119–130], 653.8[229–239] and 439.7[320–327]. The charged state distributions and corresponding molecular weights of four signature peptide candidates during LC-MS/MS analysis were well comparable with theoretic simulation data. The product ion spectra and sequences of the four peptide candidates with identified b and y ions are shown in Fig 1. Subsequently, the four peptide standards were synthesized, used for obtaining optimal mass spectrometer parameters, evaluating recovery and the matrix effect and confirming peptides in samples. The MRM parameters (fragmentor and collision energy) were acquired by individually optimizing each parameter using step values. Parent ions were doubly charged [M+2H]^2+^ ions, and fragment ions were singly charged [M+H]^+^ ions. The two fragments with highest abundance from the product ion spectra were chosen as quantitative product ions for the precursor/product ion pair (the MRM transition), preferably with a higher m/z than the m/z of the precursor ion. The sequences and corresponding product ion chosen for MRM transitions are listed in Table 1.

**Table 1:** MRM transitions on precursor and product ions of four peptide candidates.

Peptide sequence	Retention time	Precursor ion (m/z)	Product ions (m/z)	Fragmentor (V)	Collision energy(eV)
LRPVAAEIYGTK	1.296	659.3^++^	737.4^+^*/850.5^+^	125	30
GSNFQLDQLQGR	1.489	681.8^++^	716.3^+^/829.4^+^	150	25
ETTVFENLPEK	1.541	653.8^++^	876.4^+^/729.3^+^	100	20
DSALGFLR	1.802	439.7^++^	492.2^+^/605.3^+^	125	10

*: Quantitative ion

**Fig 2:**
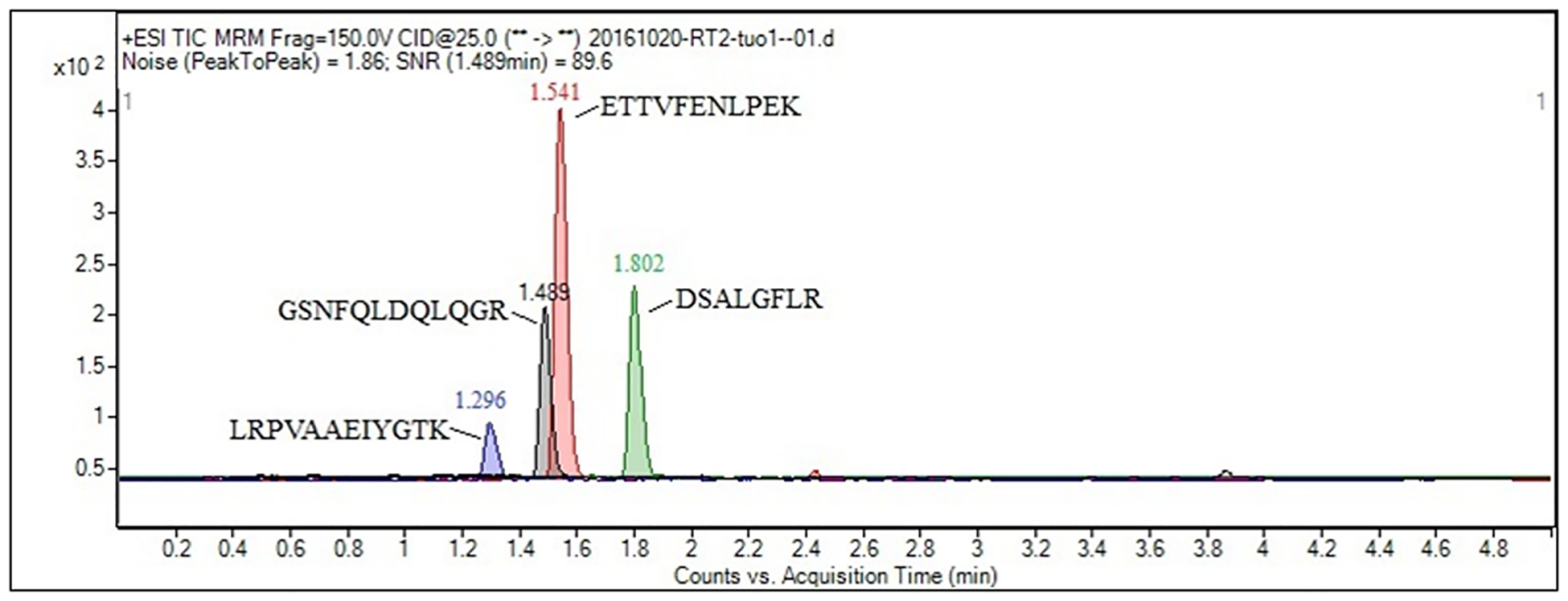
The different responses of four peptide candidates in tryptic samples.

Among the four selected tryptic peptide fragments of bovine lactoferrin, the peptide ETTVFENLPEK had the highest mass response, followed by the peptides DSALGFLR and GSNFQLDQLQGR, with the peptide LRPVAAEIYGTK trailing. The different responses of the four peptides in tryptic samples are shown in Fig 2. After comparison, the peptide ETTVFENLPEK, corresponding to residues 229–239 of bovine lactoferrin, was finally selected as the signature peptide of bovine lactoferrin for MRM assay quantification on account of its highest MS abundance, intensity and sensitivity in all the LC-MS/MS analytical samples; the remaining peptides monitored can be used for confirmatory purposes.

### Selection of an internal standard for the signature peptide

**Fig 3:**
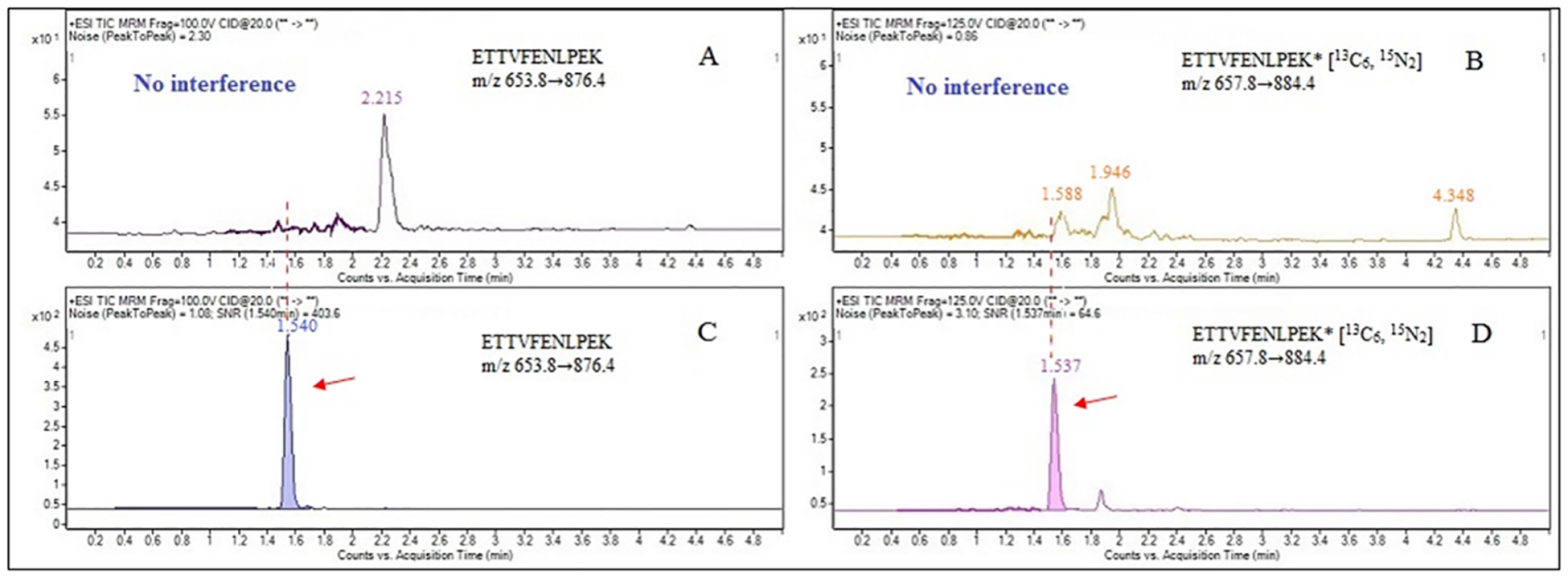
LC-MS/MS chromatograms of bovine lactoferrin signature peptide ETTVFENLPEK (C) and its corresponding SIL-IS ETTVFENLPEK* (D) in samples. No interference peaks were observed in the blank matrices (A and B).

Internal standards are often used and cautiously selected in quantitative bioanalysis for the purpose of achieving accurate, reproducible, and precise quantification. The most common IS used for protein quantification by LC-MS/MS is an SIL-analogue of the signature peptide[23]. The SIL-peptide ETTVFENLPEK* containing [^13^C_6_, ^15^N_2_] lysine (heavy peptide, molecular weight of 657.8^2+^) served as an internal standard to construct the calibration curve. The mass transitions were optimized as m/z 657.8>884.4 and m/z 657.8>737.3 from the product ion mass spectra, corresponding to y7 and y6 fragment ions, respectively. Compared with the y7 and y6 fragment ions of the signature peptide ETTVFENLPEK, the two fragment ions both showed 8 Da-shifted ions. The analysis indicated that the IS SIL-analogue of the signature peptide was also nonexistent in infant formulas before and after tryptic digestion. Furthermore, the SIL-peptide ETTVFENLPEK* had similar chromatographic behaviour to the selected signature peptide ETTVFENLPEK during the LC-MS/MS analysis (Fig 3C and 3D).

### Selection of blank matrices

Although LC-MS is a highly selective and sensitive instrument in quantitative bioanalysis, its accuracy may be changed by the diverse hydrolysis efficiencies or ionization efficiencies of the analytes in various matrices[24]. The actual enzymatic hydrolysis of the target protein in the samples can be well simulated by adding purified protein to the blank matrix. It was found that the selected signature peptide ETTVFENLPEK can quantify lactoferrin originating from sheep, goat, and buffalo milk based on the species homology of protein sequences. However, BLAST searches did not indicate any homology of the selected signature peptide ETTVFENLPEK to camel, horse, and donkey milk. Taking into account availability, camel milk powder was selected as the blank matrix and further confirmed by mass spectrometry. First, camel milk powder did not contain bovine lactoferrin and was then processed under the same conditions as the samples. Neither signature peptide ETTVFENLPEK nor SIL-peptide ETTVFENLPEK* peaks were extracted, and therefore it cannot cause interference to the quantitative detection (Fig 3A and 3B). Second, the composition of camel milk powder was relatively close to cow milk powder, and therefore can act as a blank matrix.

### Method validation

#### Specificity

Specificity of the selected peptides was evaluated by analyzing chromatographic peaks of synthetic peptides and tryptic peptides of samples. Both the signature peptide derived from digested proteins and the synthetic peptide standards showed a consistent chromatographic peak at 1.54 ± 0.02 min (Fig 3C). There were no signature peptides detected in samples without tryptic digestion. Before and after the enzymolysis of the camel milk powder by extracting MRM transitions of the signature peptides and the internal standard peptides, no peak was observed. These results showed that both the sample matrix and blank matrix had no interferences on the retention time of signature peptides.

#### Linearity, sensitivity and repeatability

The purified bovine lactoferrin added to blank matrices was used to construct a calibration curve of six concentration levels and processed as the samples were. The assay showed linear relationship over the range of 1–100 nM with a correlation coefficient (r) of 0.999 (S2 File). The obtained linear regression equation was y = 49.066x-4.7604 (n = 3). Limit of detection (LOD) and the limit of quantification (LOQ) used to evaluate the sensitivity were calculated at signal-to-noise ratio (S / N) of 10:1 and 3:1, respectively. The corresponding concentration of LOD and LOQ were 0.02 nM (0.05 mg/100 g) and 0.006 nM (0.16 mg/100 g). The experimental method had sufficient sensitivity to detect and quantify bovine lactoferrin in various infant formulas. The repeatability was assessed by calculating relative standard deviations (RSD) of multiple test results (n = 6) of samples (S2 File). The obtained RSD was 2.5–7.7% which indicated that the current LC-MS/MS method showed good reproducibility (S2 File).

#### Recovery and precision

Recovery studies were performed using standard addition of purified bovine lactoferrin to samples. Fifteen portions of premixed base powder (with a background amount of approximately 50 mg/100 g bovine lactoferrin) with the internal standard were spiked with low, intermediate and high levels of lactoferrin standards (five portions per concentration level), while three additional portions were selected as the controls. Samples were prepared according to the above mentioned processing method. The spiking recoveries were 83.1–91.6% with an RSD of 3.8–4.4%. The RSDs of intra- and inter-day precision were 1.1–4.0% and 1.5–5.4%, respectively (S2 File). The calculated results indicated that the developed method obtained a satisfying recovery and precision. The proposed LC-MS/MS approach was suitable to analyze and quantify bovine lactoferrin in infant formula.

#### Method comparison between the current method and a previous method

In Zhang's previous studies [16], determination of bovine lactoferrin based on signature peptide has been reported in MRM mode by LC-MS/MS methods. In their methods, the peptides LRPVAAEIYGTK [92–103], FFSASCVPCIDR [170–171], and VDSALYLGSR [332–341] were reported, and the peptide LRPVAAEIYGTK was selected as the signature peptide for quantification of bovine lactoferrin. A winged peptide GRDPYKLRPV*AAEI*YGTKESPQTHY (V*, Val-OH-^13^C_5_,^15^N; I*, Ile-OH-^13^C_6_,^15^N) was used as an internal standard to narrow the variations in digestion and ionization efficiency. The synthetic peptide LRPVAAEIYGTK was used for the calibration curve without biological matrices.

**Fig 4:**
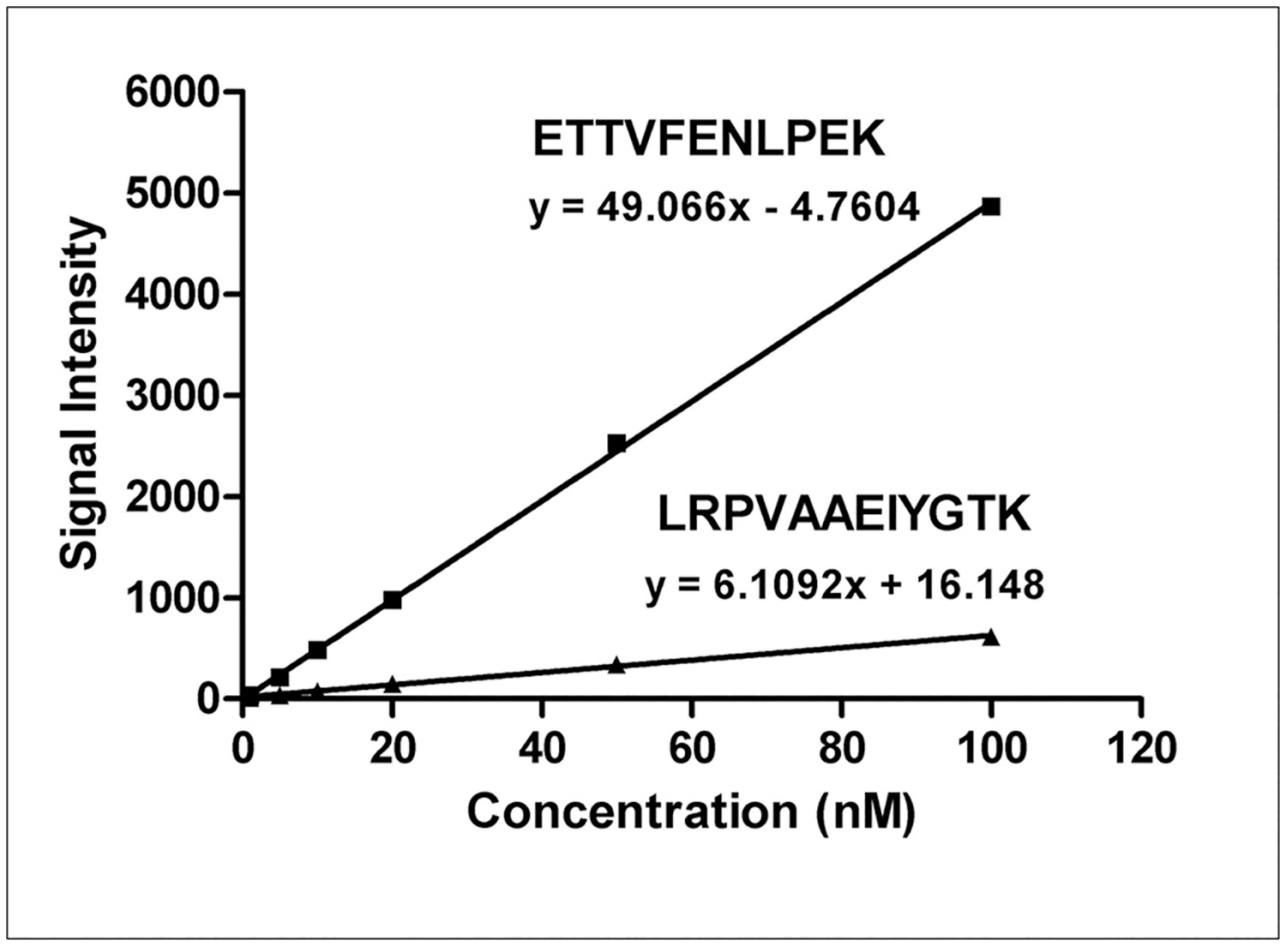
Linear responses of the bovine lactoferrin signature peptide ETTVFENLPEK in the present method and LRPVAAEIYGTK in a previous method at the same concentration range during LC-MS/MS analysis.

However, in our current method, three novel peptides GSNFQLDQLQGR (residues 120–131), ETTVFENLPEK (residues 230–240), DSALGFLR (residues 321–328) were discovered, and the peptide ETTVFENLPEK, which had the best MS response, was finally selected as signature peptide for quantification of bovine lactoferrin. The SIL-peptide ETTVFENLPEK* containing [^13^C_6_, ^15^N_2_] lysine served as an internal standard. Meanwhile, the camel milk powder was screened and selected as blank matrix. The bovine lactoferrin protein standard was available and used to establish a calibration curve. Experiments were carried out to check the advantages of the current method for quantification of bovine lactoferrin. Calibration curves were prepared using the presently selected signature peptide and Zhang's previously reported signature peptide. The analysis indicated that the two peptides were both capable of determining lactoferrin, but they obtain different MS responses in the co-eluting matrix components, as shown in Fig 4. In Zhang's paper, the LOD obtained is 0.3 mg/100 g. This clearly illustrates that the signature peptide ETTVFENLPEK we selected has better MS responses of ionization and dissociation, resulting in superior sensitivity and a lower limit of detection. Compared with synthesized peptide, protein standard exhibits exact identical behaviour as the targeted protein, which significantly minimizes the ionization efficiency and digestion variability. Therefore, the use of protein standards rather than synthesized peptides for calibration curves in blank biological matrices dramatically improves the accuracy of protein quantification based on the signature peptide by LC-MS/MS.

#### Method application

To further assess the applicability of the developed method, four popular infant formula brands from local retailers (Shenyang, China) of different stages were sampled and analyzed in the present study. All the samples were prepared according to the fore-mentioned approaches and used for LC-MS/MS analysis. The signature peptide ETTVFENLPEK of bovine lactoferrin was successfully detected in the tryptic hydrolyzates of all processed samples and the contents of bovine lactoferrin in different samples were accurately calculated (Table 2, S2 File). Of these infant formula samples, the contents of bovine lactoferrin measured were 52.60–150.56 mg/100 g, which was equivalent to a concentration of 6.8–19.5 mg/100 mL in ordinary milk, i.e., approximately 13 g of milk powder was added into 90 mL of warm water. According to the literature[25,26], human milk is rich in lactoferrin, with a colostrum concentration of 100–1600 mg/100 mL, while ordinary milk has approximately 100 mg/100 mL. The content of bovine lactoferrin in cow milk was comparatively low, with colostrum at 20–500 mg/100 mL and ordinary milk at 10 mg/100 mL. For the same stage of infant formulas, the contents of lactoferrin in infant formulas derived from different manufacturers varied widely. Additionally, the contents of bovine lactoferrin varied in different stages. Brand1, brand2 and brand3 have similar bovine lactoferrin contents approaching the level of ordinary cow milk. However, brand4 has a relatively high level content of bovine lactoferrin, probably due to a better raw milk source.

**Table 2:** Detected contents of bovine lactoferrin in different infant formulas in China by the developed LC-MS/MS method (n = 3).

Brand	Stage	Detected content (mg/100g)	Concentration (mg/100ml)
1	I	52.60	6.83
	II	79.21	10.29
	III	63.84	8.29
2	I	53.53	6.95
	II	54.13	7.03
	III	64.03	8.32
	IV	87.27	11.33
3	I	61.69	8.01
	II	64.08	8.32
	III	59.72	7.76
	IV	76.98	10.00
4	I	101.94	13.24
	II	123.35	16.02
	III	150.56	19.55
	IV	78.25	10.16

## Conclusions

In this work, three unreported signature peptides were chosen selected and identified from the tryptic peptides of bovine lactoferrin by LC-MS/MS. Among them, tryptic fragment ETTVFENLPEK with highest MS abundance, intensity and sensitivity was chosen as the signature peptide of bovine lactoferrin. An SIL-IS peptide ETTVFENLPEK* ([^13^C_6_, ^15^N_2_] lysine) has been shown to have similar analytical behaviour to the signature peptide. Protein standards added in a camel milk powder blank matrix were used to prepare a calibration curve more accurately correcting the matrix effect. In addition, the present method has also been successfully applied for analysis of lactoferrin contents in another dairy products including liquid milk, yogurt, whole milk powder, skimmed milk powder and whey protein concentrates. The application of the established method might accelerate the development of quality control and nutrient assessment of infant formulas and other dairy products containing lactoferrin.

## Supporting information

S1 File Data for selecting signature peptides for bovine lactoferrin. (DOCX)

S2 File Relevant data underlying the results described in manuscript. (XLS)
