# Supplementary material for: Selection of possible signature peptides for the detection of bovine lactoferrin in infant formulas by LC-MS/MS
Source: PLoS One. 2017 Sep 19;12(9):e0184152. doi: 10.1371/journal.pone.0184152 (PMC5604936; doi:10.1371/journal.pone.0184152)
Supplement: S1 File — (DOCX) [file pone.0184152.s001.docx]

1. Amino acid sequence of bovine lactoferrin

Gene：LTF Bos taurus (Bovine)

Length:708 Mass (Da):78,056

>sp|P24627|TRFL_BOVIN Lactotransferrin OS=Bos taurus GN=LTF PE=1 SV=2

MKLFVPALLSLGALGLCLAAPRKNVRWCTISQPEWFKCRRWQWRMKKLGAPSITCVRRAFALECIRAIAEKKADAVTLDGGMVFEAGRDPYKLRPVAAEIYGTKESPQTHYYAVAVVKKGSNFQLDQLQGRKSCHTGLGRSAGWIIPMGILRPYLSWTESLEPLQGAVAKFFSASCVPCIDRQAYPNLCQLCKGEGENQCACSSREPYFGYSGAFKCLQDGAGDVAFVKETTVFENLPEKADRDQYELLCLNNSRAPVDAFKECHLAQVPSHAVVARSVDGKEDLIWKLLSKAQEKFGKNKSRSFQLFGSPPGQRDLLFKDSALGFLRIPSKVDSALYLGSRYLTTLKNLRETAEEVKARYTRVVWCAVGPEEQKKCQQWSQQSGQNVTCATASTTDDCIVLVLKGEADALNLDGGYIYTAGKCGLVPVLAENRKSSKHSSLDCVLRPTEGYLAVAVVKKANEGLTWNSLKDKKSCHTAVDRTAGWNIPMGLIVNQTGSCAFDEFFSQSCAPGADPKSRLCALCAGDDQGLDKCVPNSKEKYYGYTGAFRCLAEDVGDVAFVKNDTVWENTNGESTADWAKNLNREDFRLLCLDGTRKPVTEAQSCHLAVAPNHAVVSRSDRAAHVKQVLLHQQALFGKNGKNCPDKFCLFKSETKNLLFNDNTECLAKLGGRPTYEEYLGTEYVTAIANLKKCSTSPLLEACAFLTR

2. Theoretical tryptic peptides and actual tryptic peptides

Forty-seven theoretical tryptic peptides of bovine lactoferrin were obtained by computational prediction using Skyline software. Among them, twenty peptides were verified after comparing in silico prediction peptides with actual tryptic peptides, which are highlighted, as shown in the following table.

| **Num** | **Peptide sequence** | **m/z** |
| --- | --- | --- |
| **1** | **K.LFVPALLSLGALGLCLAAPR.K [2, 21]** | 1026.6055++ |
| **2** | **R.WCTISQPEWFK.C [26, 36]** | 741.3477++ |
| **3** | **K.LGAPSITCVR.R [47, 56]** | 537.2922++ |
| **4** | **R.AIAEK.K [66, 70]** | 266.1605++ |
| **5** | **K.ADAVTLDGGMVFEAGR.D [72, 87]** | 804.8880++ |
| **6** | **K.LRPVAAEIYGTK.E [92, 103]** | 659.3799++ |
| **7** | **K.ESPQTHYYAVAVVK.K [104, 117]** | 796.4094++ |
| **8** | **K.GSNFQLDQLQGR.K [119, 130]** | 681.8417++ |
| **10** | **K.SCHTGLGR.S [132, 139]** | 444.2112++ |
| **11** | **K.FFSASCVPCIDR.Q [170, 181]** | 729.8289++ |
| **12** | **R.QAYPNLCQLCK.G [182, 192]** | 697.8314++ |
| **13** | **K.GEGENQCACSSR.E [193, 204]** | 677.7592++ |
| **14** | **R.EPYFGYSGAFK.C [205, 215]** | 633.2955++ |
| **15** | **K.CLQDGAGDVAFVK.E [216, 228]** | 690.3348++ |
| **16** | **K.ETTVFENLPEK.A [229, 239]** | 653.8299++ |
| **17** | **R.DQYELLCLNNSR.A [243, 254]** | 762.8592++ |
| **28** | **R.APVDAFK.E [255, 261]** | 374.2054++ |
| **19** | **K.ECHLAQVPSHAVVAR.S [262, 276]** | 837.4307++ |
| **20** | **R.SVDGK.E [277, 281]** | 253.1345++ |
| **21** | **K.EDLIWK.L [282, 287]** | 402.2185++ |
| **22** | **R.SFQLFGSPPGQR.D [303, 314]** | 660.8384++ |
| **23** | **R.DLLFK.D [315, 319]** | 318.1918++ |
| **24** | **K.DSALGFLR.I [320, 327]** | 439.7402++ |
| **25** | **K.VDSALYLGSR.Y [332, 341]** | 540.7878++ |
| **26** | **R.YLTTLK.N [342, 347]** | 369.7234++ |
| **27** | **R.ETAEEVK.A [351, 357]** | 403.2005++ |
| **28** | **R.VVWCAVGPEEQK.K [363, 374]** | 701.3452++ |
| **29** | **K.GEADALNLDGGYIYTAGK.C [405, 422]** | 914.4416++ |
| **30** | **K.CGLVPVLAENR.K [423, 433]** | 614.3293++ |
| **31** | **K.HSSLDCVLRPTEGYLAVAVVK.K [438, 458]** | 1157.6148++ |
| **32** | **K.ANEGLTWNSLK.D [460, 470]** | 616.8171++ |
| **33** | **K.SCHTAVDR.T [474, 481]** | 473.2140++ |
| **34** | **R.LCALCAGDDQGLDK.C [519, 532]** | 768.3451++ |
| **35** | **K.CVPNSK.E [533, 538]** | 352.6734++ |
| **36** | **K.YYGYTGAFR.C [541, 549]** | 549.2562++ |
| **37** | **R.CLAEDVGDVAFVK.N [550, 562]** | 711.8503++ |
| **38** | **K.NDTVWENTNGESTADWAK.N [563, 580]** | 1019.4429++ |
| **39** | **R.LLCLDGTR.K [589, 596]** | 474.2526++ |
| **40** | **R.KPVTEAQSCHLAVAPNHAVVSR.S [597, 618]** | 1186.1186++ |
| **41** | **R.AAHVK.Q [622, 626]** | 263.1608++ |
| **42** | **K.QVLLHQQALFGK.N [627, 638]** | 691.4012++ |
| **43** | **K.NCPDK.F [642, 646]** | 317.1367++ |
| **44** | **K.FCLFK.S [647, 651]** | 357.6858++ |
| **45** | **K.NLLFNDNTECLAK.L [656, 668]** | 776.3772++ |
| **46** | **K.LGGRPTYEEYLGTEYVTAIANLK.K [669, 691]** | 1279.6605++ |
| **47** | **K.CSTSPLLEACAFLTR.- [693, 707]** | 863.4186++ |

3. Product ion spectrums for the verified twenty peptides


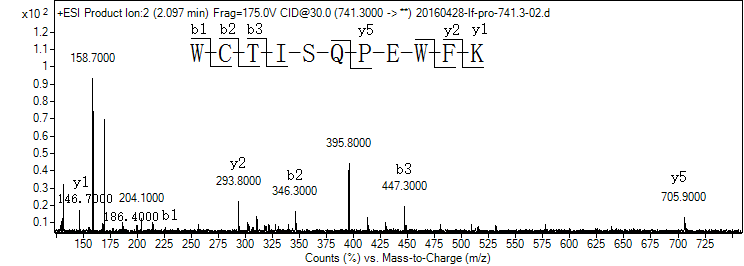


(1)


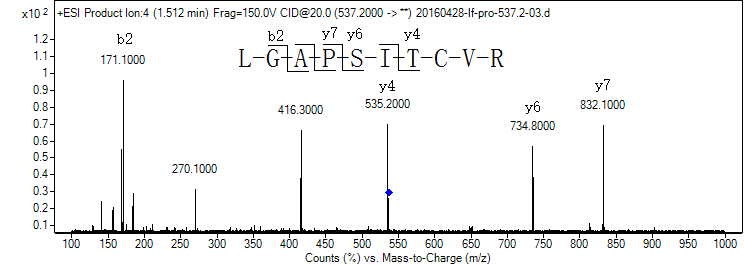


(2)


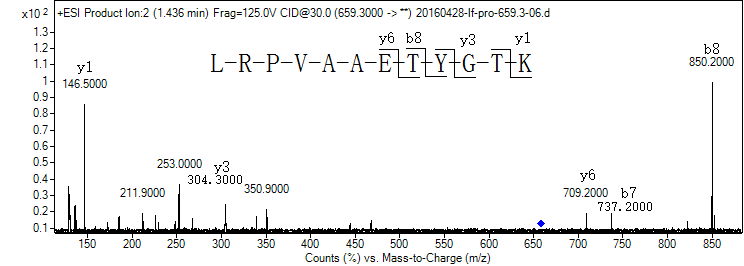


(3)


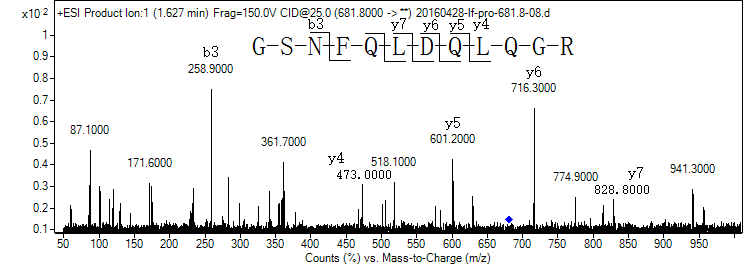


(4)


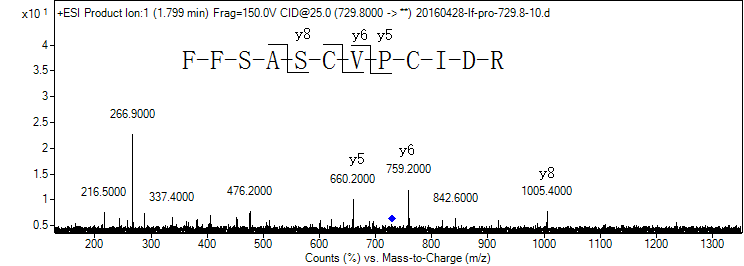


(5)


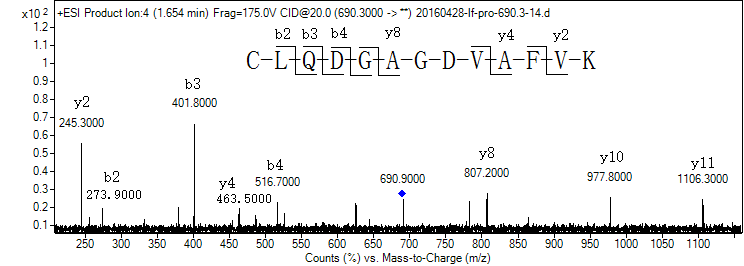


(6)


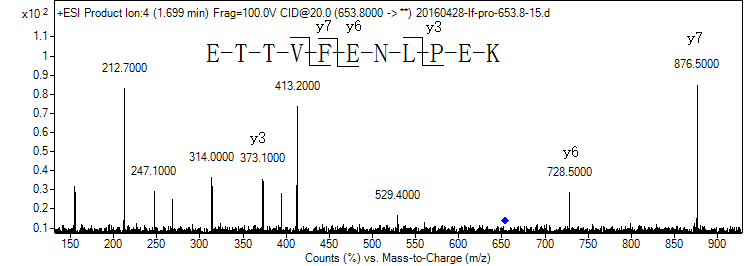


(7)


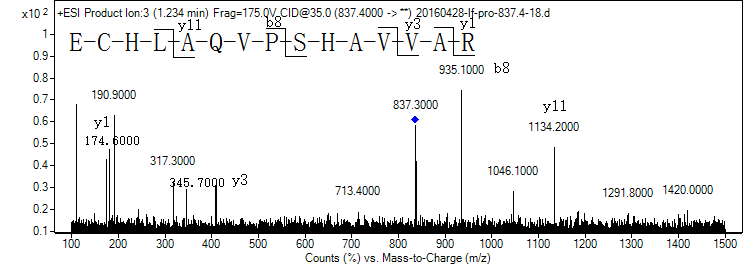


(8)


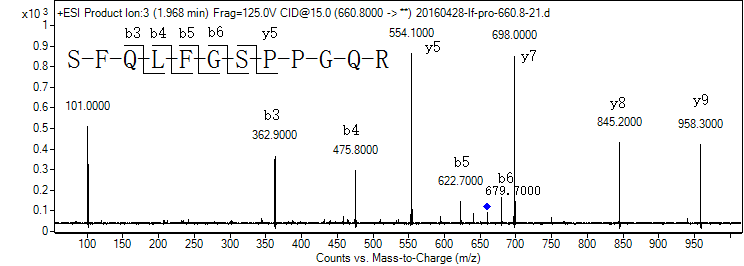


(9)


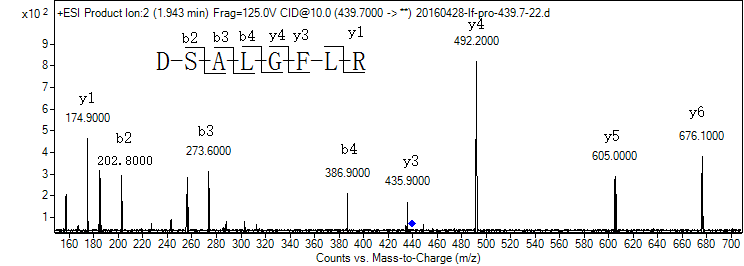


(10)


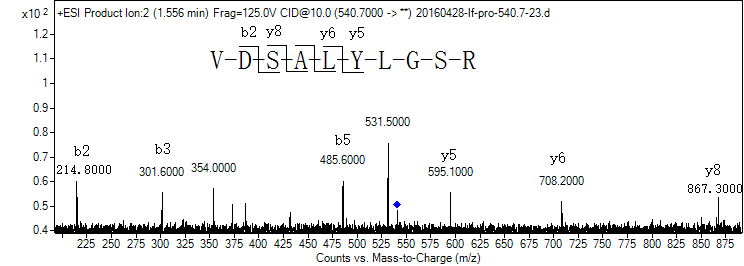


(11)


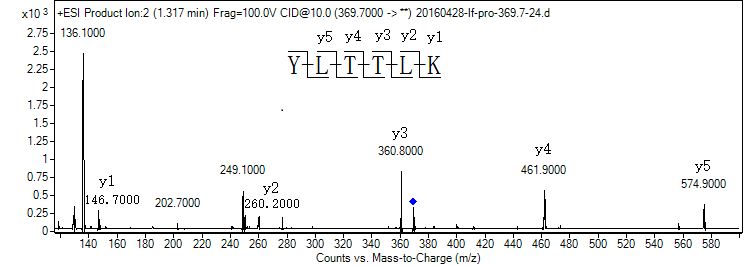


(12)


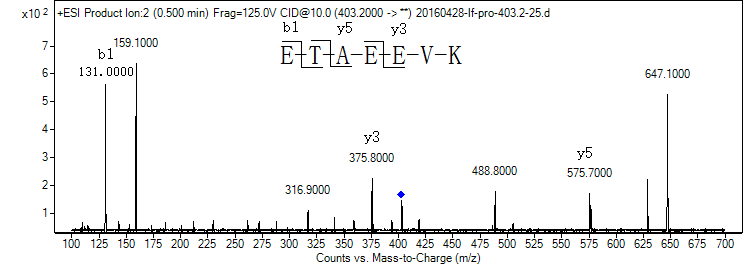


(13)


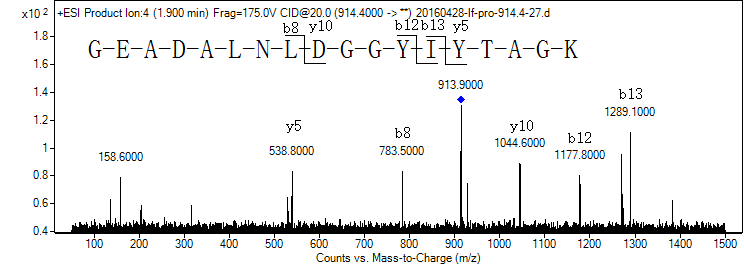


(14)


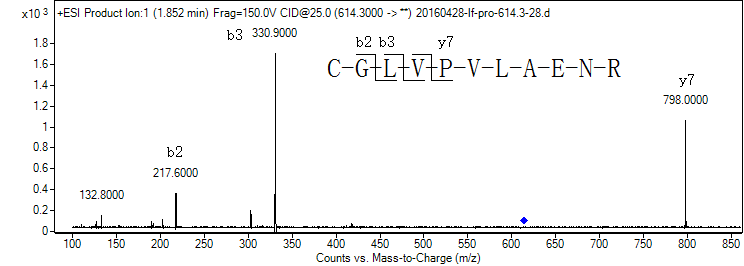


(15)


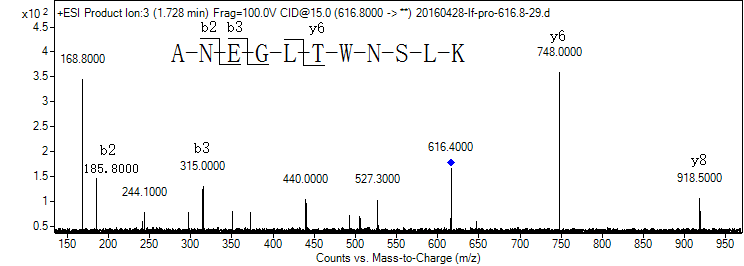


(16)


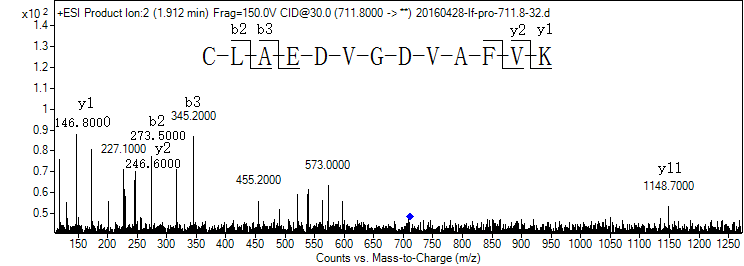


(17)


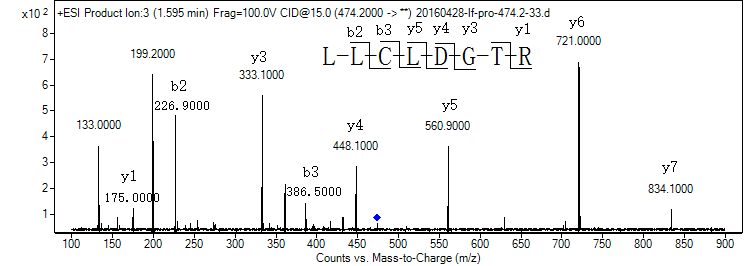


(18)


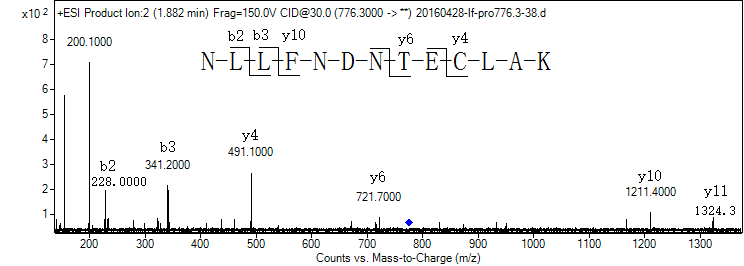


(19)


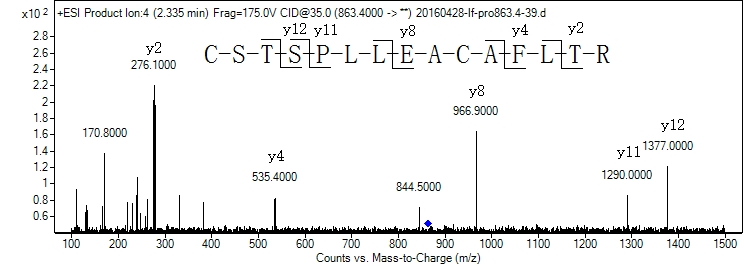


(20)
